# Characterising Australian memory clinics: current practice and service needs informing national service guidelines

**DOI:** 10.1186/s12877-022-03253-7

**Published:** 2022-07-14

**Authors:** Sharon L. Naismith, Johannes C. Michaelian, Lee-Fay Low, Valerie Arsenova, Inga Mehrani, Katrina Fyfe, Nicole A. Kochan, Susan E. Kurrle, Christopher Rowe, Perminder S. Sachdev

**Affiliations:** 1 School of Psychology, Charles Perkins Centre and the Brain and Mind Centre, University of Sydney, Sydney, Australia; 2 Healthy Brain Ageing Program, Brain and Mind Centre, The University of Sydney, 94 Mallett Street, Camperdown, NSW 2050 Australia; 3 Faculty of Medicine and Health, University of Sydney, Sydney, Australia; 4 Centre for Healthy Brain Ageing (CHeBA), School of Psychiatry, University of New South Wales, Sydney, Australia; 5 School of Health and Medical Sciences, University of Western Australia, Perth, Australia; 6 Department of Molecular Imaging and Therapy, Austin Health, The University of Melbourne, Melbourne, Victoria Australia; 7 Neuropsychiatric Institute, Prince of Wales Hospital, Randwick, Australia

**Keywords:** Memory clinics (MCs), Current clinical practice, Dementia, Staffing, Referrals, Case conference, Feedback, Care plan development, Post-diagnostic care, Care plan implementation, Best practice

## Abstract

**Background:**

Memory clinics (MCs) play a key role in accurate and timely diagnoses and treatment of dementia and mild cognitive impairment. However, within Australia, there are little data available on current practices in MCs, which hinder international comparisons for best practice, harmonisation efforts and national coordination. Here, we aimed to characterise current service profiles of Australian MCs.

**Methods:**

The ‘Australian Dementia Network Survey of Expert Opinion on Best Practice and the Current Clinical Landscape’ was conducted between August-September 2020 as part of a larger-scale Delphi process deployed to develop national MC guidelines. In this study, we report on the subset of questions pertaining to current practice including wait-times and post-diagnostic care.

**Results:**

Responses were received from 100 health professionals representing 60 separate clinics (45 public, 11 private, and 4 university/research clinics). The majority of participants were from clinics in metropolitan areas (79%) and in general were from high socioeconomic areas. While wait-times varied, only 28.3% of clinics were able to offer an appointment within 1-2 weeks for urgent referrals, with significantly more private clinics (58.3%) compared to public clinics (19.5%) being able to do so. Wait-times were less than 8 weeks for 34.5% of non-urgent referrals. Only 20.0 and 30.9% of clinics provided cognitive interventions or post-diagnostic support respectively, with 7.3% offering home-based reablement programs, and only 12.7% offering access to group-based education. Metropolitan clinics utilised neuropsychological assessments for a broader range of cases and were more likely to offer clinical trials and access to research opportunities.

**Conclusions:**

In comparison to similar countries with comprehensive government-funded public healthcare systems (i.e., United Kingdom, Ireland and Canada), wait-times for Australian MCs are long, and post-diagnostic support or evidence-based strategies targeting cognition are not common practice. The timely and important results of this study highlight a need for Australian MCs to adopt a more holistic service of multidisciplinary assessment and post-diagnostic support, as well as the need for the number of Australian MCs to be increased to match the rising number of dementia cases.

**Supplementary Information:**

The online version contains supplementary material available at 10.1186/s12877-022-03253-7.

## Introduction

While there is no consensus on the definition of a memory clinic (MC), nor recommendations on their composition, services offered and standards [1] – MCs can be broadly described as a multidisciplinary medical assessment centre that specialises in the assessment and treatment of dementia and cognitive decline [2, 3]. MCs play a key role in early, accurate and timely diagnosis of dementia and identification of mild cognitive impairment (MCI). With their integrated multidisciplinary focus, MCs are also paramount for treatment and care-planning purposes [3, 4] and maintenance of independent functioning, which in turn can improve prognosis and delay admission to residential care [5–7]. Such an approach is likely to be cost-effective [8] and associated with improvement in quality of life [9] for the person with dementia and their carers [10]. In addition, if MCI is identified early, risk reduction strategies can be implemented, optimising cognition, and thereby workplace and community productivity. It further improves social engagement [11], potentially delaying the onset of dementia [12].

There is a dearth of contemporary data regarding MCs in Australia. Of the limited available data it seems that wait-times for Australian publicly-funded MCs are around 12 weeks, with the vast majority having “catchment restrictions” in place [2]. This is considerably longer than observed in the United Kingdom, Ireland or Canada, where wait-times of three to five weeks are reported, and a waiting period of less than six weeks is considered ideal [13–15]. Compounding this problem, there is no standard pathway for a dementia diagnosis, while service provision and care pathways are poorly coordinated and lack harmonisation within and across Australian states and territories [16, 17].

Of significance, available data suggests that very few Australian MCs provide ongoing post-diagnostic care, coordination of care or risk reduction programs. In a recent survey of 90 Australian public and private sector service providers [2], less than one third reported providing *any* kind of post-diagnostic support. Of those that did, 52% provided only one session. Limited resourcing and lack of training were often cited as reasons for not providing cognitive and other non-pharmacological interventions. The general lack of post-diagnostic support for people with dementia has been highlighted in several reviews [18], yet Australian MCs focus largely on diagnostics and are not typically funded to provide interventions.

Internationally, there are national frameworks for MC services. For example, the Royal College of Psychiatrists in the United Kingdom has established the Memory Services National Accreditation Program (MSNAP) [19], which includes comprehensive guidelines as well as an audit and accreditation process [20]. It outlines clear benchmarks on management, referrals and access, assessment and diagnosis, ongoing care and follow-up, as well as pharmacological and psychosocial interventions. In the decade it has been running, services have increased caseloads whilst also achieving reduced wait-times [21]. Inline with increased caseloads, the number of staff working in the UK MCs has also increased over the past decade.

**Fig. 1:**
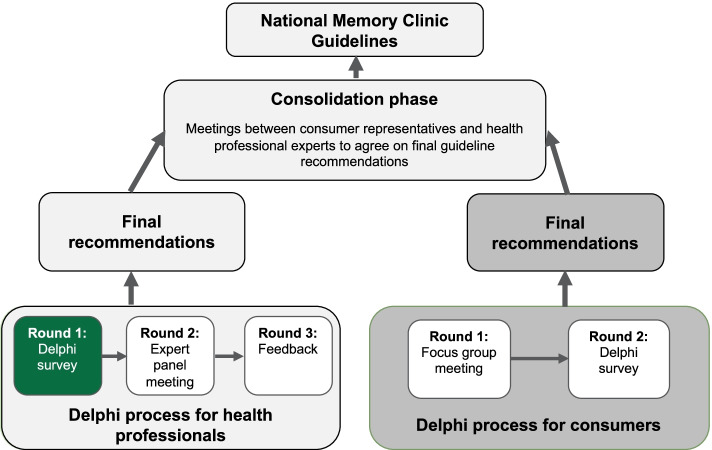
National Memory Clinic Guidelines development flow chart

The lack of national MC standards in Australia is problematic for consumers seeking early and accurate diagnoses and care [16, 19]. Additionally, this may preclude federal and state-based funding opportunities for enhanced support and services. The Australian Dementia Network (ADNeT) seeks to harmonise and facilitate access to MC services nationwide and improve diagnostic and care standards. This paper reports on a subset of questions pertaining to current practice from the survey ‘Australian Dementia Network Survey of Expert Opinion on Best Practice and the Current Clinical Landscape’ (Australian Dementia Network survey) conducted between August-September 2020 as part of a larger-scale Delphi process deployed to develop national MC Service Guidelines (Fig. 1).

## Methods

### Sample and setting

The sampling frame (for the Australian Dementia Network survey) included any Australian clinician or coordinator who self-identified as working for a specialised cognitive assessment service for cognitive decline and dementia and/or having a professional interest in MCs. Several recruitment strategies were employed. First, a single email invitation to participate in the survey was sent to the official contacts of all clinics previously engaged with the ADNeT-Memory Clinics initiative. All clinicians were asked to forward the survey to their clinical team or other interested colleagues. Two reminder emails were sent. In addition, the survey was advertised via social media, such as the ADNeT Twitter account, ADNeT webinars, and within professional networks and organisations (i.e., College of Clinical Neuropsychologists, Dementia Australia, Australian & New Zealand Society for Geriatric Medicine). The survey was open from the 4th of August until the 16th of September 2020.

### Survey and procedure

The ‘Australian Dementia Network Survey of Expert Opinion on Best Practice and the Current Clinical Landscape’ was delivered in Qualtrics [22]. This survey comprised Stage 1 of a Delphi process seeking health professional input into the development of National Memory Clinic Service Guidelines. The conduct of this study was approved by UNSW’s Human Research Ethics Approval Panel (HREAP) D: Biomedical (reference: HC200394). All potential respondents provided informed consent electronically before the start of the survey, and all study methods were conducted in compliance with the Helsinki Declaration. The survey focused on ‘current’ and ‘ideal’ clinical practices, comprising 12 sections: 1) staffing; 2) clients; 3) referrals; 4) prioritisation; 5) confidentiality/consent; 6) assessment; 7) diagnosis; 8) case conference; 9) feedback; 10) care plan development; 11) post-diagnostic care and care plan implementation; and 12) clients with diverse needs. All questions were structured, requiring the respondent to choose from a finite list of responses (e.g., “agree/disagree”, “yes/no”) utilising a mixture of multiple-choice “checkbox” or single-choice “radio buttons” answer options. Here, we report on current practices only from a health professional’s perspective. Importantly, any client needs identified or commented on are from a health professional perspective. A copy of the full survey can be acquired from the corresponding author upon request.

### Data analysis

All survey responses were recorded and initially saved in Qualtrics [22]. Data were exported into SPSS Version 25 (SPSS Inc., IBM Corp. in Armonk, NY, USA), where all statistical analyses were conducted. Descriptive analyses (i.e., frequencies and percentages) were performed to provide an overview of the current clinical landscape, with Chi-square tests (χ^2^) conducted to compare different proportions between different groups for each categorical variable. All Chi-square tests included one degree of freedom. All analyses employed an α level of 0.05.

Missing or ‘unable to comment’ responses were recorded, and the total number of ‘valid responses’ became the denominator for that item. To accurately represent the data from clinics with more than one respondent, we only included responses from one respondent per clinic for the following variables: wait-times, consent and disclosure, assessment and diagnosis, client feedback, post-diagnostic practices and service improvements. For these variables, responses were utilised from the following clinicians, in order and according to availability of data: a) clinic coordinator, b) registered nurse, c) medical specialist (e.g., neurologist, geriatrician), d) allied health professionals (e.g., neuropsychologist, occupational therapist).

Respondents self-identified as working in a ‘metropolitan’ or ‘regional’ setting and provided the post code of their clinic. The Index of Relative Socio-Economic Advantage and Disadvantage (IRSAD) was utilised to calculate the economic and social conditions prevalent within a particular clinic’s postcode [23]. The IRSAD is split into 10 equal deciles, ranging from a score of ‘1’ (lowest socio-economic conditions) to ‘10’ (highest average socio-economic conditions).

## Results

### Respondents

Responses were received from 100 health professionals working in 60 separate clinics (i.e., there were multiple responses received from 21 clinics). The 60 clinics included 45 publicly funded clinics (PUB-Cs), 11 private sector services (PRIV-Cs) and 4 hybrid university/research clinics (i.e., clinics embedded within universities, funded via grants with additional use of Medicare), which were coded as ‘private’ clinics for these purposes. From these 60 clinics identified, 54 were located in different postcodes. The majority of respondents were located in metropolitan areas (79%). Seventy six percent of respondents were from PUB-Cs. New South Wales accounted for 43% of the respondents and Victoria for 39%. Based on the median IRSAD score, the economic and social conditions were considered to be very high (median = 9.0; mean = 7.8, range: 1 – 10) for all clinics included in this survey.

Respondents were neuropsychologists (40%), geriatricians (25%), clinical coordinators/administrative support (14%), neurologists (10%), registered nurses (9%), and other allied health professionals (i.e., occupational therapist, speech pathologist) (7%). There were 28 male respondents, 66 female, and six unknown. Table 1 shows the characteristics of respondents. The proportion of respondents in administrative/clinical coordination roles (relative to clinician roles) was significantly higher in regional compared to metropolitan regions (28.6% vs. 10.1%) (χ^2^ = 4.7, *p* = .03). Mobile services were offered by 15% (*n* = 9/60) of clinics, whilst 80% (*n* = 48/60) offered telehealth. While there were no differences between metropolitan and regional clinics in terms of telehealth provision, mobile services were significantly more likely to be offered by regional clinics compared to metropolitan clinics (75% vs. 0%) (χ^2^ = 14.2, *p* = .0002).

**Table 1:** Characteristics of survey respondents

	Overall	Metropolitan	Regional	Public	Private	NSW	VIC	QLD	SA	WA	TAS
**Respondents, n (%)**	100 (100.0)	79 (79.0)	21 (21.0)	76 (76.0)	24 (24.0)	43 (43.0)	39 (39.0)	7 (7.0)	4 (4.0)	4 (4.0)	3 (3.0)
**Profession, n (%)**											
Clinical Coordinator/ Administrative support	14 (14.0)	8 (10.1)	6 (28.6)^*^	10 (13.2)	4 (16.7)	6 (14.0)	8 (20.5)	0 (0.0)	0 (0.0)	0 (0.0)	0 (0.0)
Clinical Neuropsychologist	40 (40.0)^$^	37 (46.9)^$^	3 (14.3)^*^	27 (35.5)	13 (54.2)	19 (44.2)	14 (35.9)	3 (42.9)^$^	3 (75.0)	1 (25.0)	0 (0.0)
Psychiatrist	2 (2.0)	2 (2.5)	0 (0.0)	2 (2.6)	0 (0.0)	2 (4.7)	0 (0.0)	0 (0.0)	0 (0.0)	0 (0.0)	0 (0.0)
Neurologist	10 (10.0)	9 (11.4)	1 (4.8)	7 (9.2)	3 (12.5)	3 (7.0)	4 (10.3)	0 (0.0)	1 (25.0)	1 (25.0)	1 (33.3)
Geriatrician	25 (25.0)	17 (21.5)	8 (38.1)	19 (25.0)	6 (25.0)	12 (27.9)	6 (15.4)	4 (57.1)	0 (0.0)	1 (25.0)	2 (66.7)
Registered Nurse	9 (9.0)	5 (6.3)	4 (19.0)	8 (10.5)	1 (4.2)	2 (4.7)	6 (15.4)	0 (0.0)	0 (0.0)	1 (25.0)	0 (0.0)
Allied health (i.e., Occupational therapist, speech pathologist)	7 (7.0)	6 (7.6)	1 (4.8)	7 (9.2)	0.0 (0.0	0 (0.0)	7 (17.9)**	0 (0.0)	0 (0.0)	0 (0.0)	0 (0.0)
Other	13 (13.0)	8 (10.1)	5 (23.8)	10 (13.2)	3 (12.5)	8 (18.6)	3 (7.7)	1 (14.3)	0 (0.0)	1 (25.0)	0 (0.0)
**Clinic setting, Public/Private, (Public)**	83/17 (83.0)	65/14 (82.3)	18/3 (85.7)	–	–	31/12 (72.1)	38/1 (97.4)**	7/0 (100.0)	3/1 (75.0)	2/2 (50.0)	2/1 (66.7)
**Locality Metropolitan/Regional, (Metropolitan)**	79/21 (79.0)	–	–	59/17 (77.6)	20/4 (83.3)	33/10 (76.7)	32/7 (82.1)	6/1 (85.7)	4/0 (100.0)	3/1 (75.0)	1/2 (33.3)

^$^One respondent identified as a ‘clinical psychologist’

*Significant difference between metropolitan versus regional (Chi-square; *p* < .05)

**Significant difference between NSW versus VIC (Chi-square; *p* < .01)

### Justification for immediate assessment (*n* = 95)

For 95 respondents, an **immediate assessment need** was considered to be justified when the presenting problem was identified as: safety concerns in the current living situation (for client or family/carer) (91.6%); suspected self-neglect or abuse (83.2%); clients who care for others (e.g., parents of young children, carers of a person with disability) (81.1%); rapid decline of cognitive and/or memory functions (76.8%); significant carer burden and stress (74.7%); other safety concerns (e.g., driving) (69.5%); sudden and marked onset of cognitive symptoms (58.9%); clients with a suspected diagnosis of young onset dementia (47.4%); and guardianship issues (41.1%).

### Wait-times (*n* = 55)

Tables 2 and 3 shows wait-times for all clinics by service and region. For clients identified as having an **immediate assessment need** (i.e., priority clients), 28.3% of clinics noted they were able to offer a first appointment within 1-2 weeks, and 32.1% of clinics within 3-4 weeks. A significant difference was observed between the PUB-Cs (19.5%, *n* = 8/41) and PRIV-Cs (58.3%, *n* = 7/12) clinics’ capacity to provide an initial appointment within 1-2 weeks (χ^2^ = 6.8, *p* = .01).

**Table 2:** Current wait-times for clients with an immediate assessment need (% agreement)

	Overall (*n* = 53)	Metro (*n* = 38)	Regional (*n* = 15)	Public (*n* = 41)	Private (*n* = 12)
Within 1-2 weeks	28.3	31.6	20.0	19.5^**^	58.3^**^
Within 3-4 weeks	32.1	31.6	33.3	31.7	33.3
Within 2 months	26.4	26.3	26.7	31.7	8.3
Within 3 months	11.3	7.9	20.0	14.6	0.0
> 3 months	1.9	2.6	0.0	2.4	0.0

**Significant difference (Chi-square; *p* < .01)

For referrals **without an immediate assessment need** (Table 3), 34.5% of clinics could provide an initial appointment within 2 months of referral, while the remaining 65.5% reported longer wait-times. A larger proportion of PRIV-Cs were able to provide an initial appointment within 1 month, compared to PUB-Cs (38.5% vs. 4.8% respectively: χ^2^ = 5.2, *p* = .002) and 2 months (69.3% vs. 23.8% respectively: χ^2^ = 8.9, *p* = .003).

**Table 3:** Current wait-times for clients without an immediate assessment need (% agreement)

	Overall (*n* = 55)	Metropolitan (*n* = 40)	Regional (*n* = 15)	Public (*n* = 42)	Private (*n* = 13)
Within 1 month	12.7	15.0	6.7	4.8^**^	38.5^**^
Within 2 months	21.8	25.0	13.3	19.0	30.8
Within 3 months	38.2	37.5	40.0	42.9	23.1
Within 6 months	20.0	17.5	26.7	23.8	7.7
6-12 months	3.6	2.5	6.7	4.8	0.0
Longer than 12 months	3.6	2.5	6.7	4.8	0.0

** Significant difference between public and private (Chi-square; *p* < .01)

### Consent and disclosure of results (*n* = 93)

Regarding capacity for consent, respondents agreed that it should be sought a) if the client’s capacity to consent changes (e.g., based on clinician or family impression) (54.8%); b) if the treatment/ care plan changes (48.4%); c) if the client, family/carer, or medical professional request it (48.4%); d) at the initial assessment for some clients (43.0%); and, e) at regular intervals (25.8%). While 41.9% of respondents felt an assessment of the client’s capacity to consent should be conducted at the initial assessment for all clients, this was more common in regional (65.0%, *n* = 13/20) compared to metropolitan (35.6%, *n* = 26/73) clinics (χ^2^ = 5.4, *p* = .02).

Out of 99 respondents, there was high agreement that clients should be asked if they wish to know the diagnosis (85.9%), and that clients should be asked if and with whom the outcome of the assessment should be shared (96.0%). Similarly, 91.9% agreed that an interview with the client must be arranged in a manner that ensures that the client’s wishes and needs are identified independently from others. Ninety one percent of respondents agreed that an interview with someone who knows the client well (informant) must be arranged. Agreement for this statement was more likely amongst metropolitan (*n* = 71/75) compared to regional respondents (*n* = 17/21) (94.7% vs. 81.0% respectively) (χ^2^ = 4.1, *p* = .04) and PUB-Cs (94.7%, *n* = 71/75) compared to PRIV-Cs (79.2%, *n* = 19/24) clinics (χ^2^ = 4.5, *p* = .03).

If a client did not wish to know the diagnosis, there was almost unanimous agreement amongst the 97 respondents (95.9%) that the staff must respect that wish. The majority of respondents (77.3%) agreed that if the family /carer requests that the diagnosis is not disclosed (and the client cannot consent) it should be initially respected. However, 96.9% (*n* = 95/98) agreed that this decision should be further discussed over time.

### Current assessment and diagnostic practices (*n* = 97)

Clinical assessments and inter-disciplinary case conference data are provided in Supplementary Material 1. Notably, significantly more PUB-Cs used a consensus diagnosis model and were more likely to discuss the client’s legal and financial capabilities, compared to PRIV-Cs. Legal issues were covered significantly less in both metropolitan and PRIV-Cs, compared to regional and PUB-Cs, respectively. Metropolitan clinics were significantly more likely to discuss imaging results, pharmacological intervention options and suitability for industry-sponsored clinical trials, compared to regional clinics.

#### Neuropsychological assessment

The frequencies of all reasons identified for conducting neuropsychological assessments are provided in Table 4. Diagnostic uncertainty/ differential diagnosis was most common (> 90% respondents), followed by complex or unusual symptom pattern, functional decline despite ‘normal’ scores on gross screening tools, pronounced language difficulties, the need to understand cognitive profile to inform treatment and management, expected young onset dementia, pronounced behavioural changes (e.g., suspected behavioural frontotemporal dementia), capacity for decision making (e.g., financial, treatment, care plan, placement etc.), and subtle cognitive changes (e.g., subjective memory complaint or mild cognitive impairment).

**Table 4:** Frequencies for all reasons identified for conducting neuropsychological assessments (% agreement)

	Overall (*n* = 97)
Diagnostic uncertainty / differential diagnosis	91.8
Complex or unusual symptom pattern	85.6
Functional decline despite ‘normal’ scores on gross screening tools	82.5
Pronounced language difficulties (e.g., suspected primary progressive aphasia)	81.4
Expected young onset dementia	80.4
Need to understand cognitive profile to inform treatment and management	80.4
Pronounced behavioural changes (e.g., suspected behavioural frontotemporal dementia)	79.4
Capacity for decision making (e.g., financial, treatment, care plan, placement etc.)	72.2
Subtle cognitive changes (e.g., subjective memory complaint or mild cognitive impairment)	70.1
Rapid decline	45.4
New onset psychiatric disorder (e.g. late-onset depression, psychosis)	34.0
Substance induced	26.8
Every Memory Clinic client	11.3
Other	6.2

Respondents from PRIV-Cs were significantly more likely to agree that a neuropsychological assessment should be provided for every client, when compared to PUB-Cs (25.0%, *n* = 6/24 vs. 6.8%, *n* = 5/73, respectively: χ^2^ = 5.7, *p* = .02). Moreover, compared to regional respondents, more metropolitan respondents agreed that neuropsychological assessment should be sought in the following cases: diagnostic uncertainty / differential diagnosis (94.8%, *n* = 73/77 vs. 80.0% *n* = 16/20: χ^2^ = 4.5, *p* = .03); rapid decline (50.6%, *n* = 39/77 vs. 25.0%, *n* = 5/20: χ^2^ = 5.7, *p* < .0001); complex or unusual symptom pattern (89.6%, *n* = 69/77 vs. 70.0%, *n* = 14/20: χ^2^ = 4.3, *p* = 0.04); and capacity for decision making (e.g., financial, treatment, care plan, placement) (77.9%, *n* = 60/77 vs. 50.0%, *n* = 10/20: χ^2^ = 6.1, *p* = .01).

### Client feedback

As shown in Tables 5 and 6, formal feedback to clients was provided in 81.8% of clinics, with 71.4% of all clinics offering a separate feedback session to the client about the outcomes of assessment. Forty clinics provided timelines for clinic feedback, which was within 2 weeks or 4 weeks by 30 and 35% of clinics, respectively, and 15 and 20% provided feedback more than 4 weeks after assessment or other timeframes, respectively. Information most commonly (> 70%) provided by clinics (*n* = 51) included: diagnosis, driving, pharmacological options, lifestyle changes, next steps for post-diagnostic care planning, Dementia Australia, community services, risk management, further medical assessments, carer support, safety, and impairment management. Less commonly provided (< 70%) was information on cognitive interventions, legal advice services, research participation opportunities, pension or benefits, suicidal risk, and brain bank. Significantly more PUB-Cs provided information on carer support than PRIV-Cs (86.8%, *n* = 33/38 vs. 61.5%, *n* = 8/13, respectively: χ^2^ = 3.9, *p* = .05).

**Table 5:** Current wait-times for the provision of a feedback session (% agreement)

	Overall (*n* = 40)	Metro (*n* = 30)	Regional (*n* = 10)	Public (*n* = 33)	Private (*n* = 7)
2 weeks after assessment	30.0	33.3	20.0	24.2	57.1
4 weeks after assessment	35.0	30.0	50.0	42.4^**^	0.0^**^
> 4 weeks after assessment	15.0	16.7	10.0	12.1	28.6
Other	20.0	20.0	20.0	21.2	14.3

** Significant difference (Chi-square; *p* < .01)

**Table 6:** Topics that are currently addressed (if applicable) in the feedback session with the client and/or family/carer (% agreement)

	Overall (***n*** = 51)	Metro (***n*** = 38)	Regional (***n*** = 13)	Public (***n*** = 38)	Private (***n*** = 13)
Diagnosis	98.0	100.0	92.3	100.0	92.3
Driving	98.0	97.4	100.0	97.4	100.0
Pharmacological intervention options/ recommendations	96.1	97.4	92.3	94.7	100.0
Next steps for post-diagnostic care planning	94.1	94.7	92.3	94.7	92.3
Beneficial lifestyle changes	94.1	92.1	100.0	94.7	92.3
Information on Dementia Australia	88.2	86.8	92.3	86.8	92.3
Information on community care services	86.3	84.2	92.3	89.5	76.9
Risk management	84.3	78.9	100.0	84.2	84.6
Further medical assessments	80.4	84.2	69.2	76.3	92.3
Carer support	80.4	76.3	92.3	86.8^*^	61.5^*^
Safety issues	80.4	78.9	84.6	78.9	84.6
Impairment management	70.6	73.7	61.5	71.1	69.2
Cognitive intervention options	60.8	60.5	61.5	60.5	61.5
Legal advice services	52.9	52.6	53.8	55.3	46.2
Research participation opportunities	51.0	57.9	30.8	55.3	38.5
Pension and benefits	39.2	39.5	38.5	42.1	30.8
Suicidal risk	35.3	34.2	38.5	34.2	38.5
Information on brain bank	9.8	10.5	7.7	5.7	23.1
Other	2.0	2.6	0.0	2.6	0.0

*Significant difference (Chi-square; *p* < .05)

### Post-diagnostic practices (*n* = 55)

- Pharmacological post-diagnostic care (*n* = 49): The majority of clinics provided medication review (e.g., medications with anticholinergic effects) (93.9%) and prescribed cholinesterase inhibitors (93.9%) and/or memantine (87.8%). The majority (83.7%) offered pharmacological interventions for psychological/ psychiatric symptoms, and 81.6% for dementia symptoms. No differences between metropolitan and regional, or PUB-Cs and PRIV-Cs were observed.
- Provision of non-pharmacological post-diagnostic care (*n* = 55): Carer/family support was reported by 76.4% of clinics, with 40% of clinics providing family education and counselling. Management of depression was provided by 69.1% of clinics. Development of detailed care plans and advance care directives occurred in 67.3 and 32.7% of clinics, respectively. Provision of risk reduction education, telehealth, and behavioural management intervention were reported by 60.0, 54.5, and 54.5% of clinics, respectively. Only 52.7% of the clinics reported providing non-pharmacological interventions to support daily, social, and occupational functioning.
- Cognitive interventions (*n* = 55): Memory strategy training was provided by 20.0% of clinics, with only 14.5% of clinics providing at least two sessions of cognitive interventions, including memory strategy training. Only two (3.6%) provided telehealth memory rehabilitation; and only one (1.8%) provided computerised cognitive training. Group-based programs focused on cognition and wellbeing were provided by 12.7%. Other unspecified post-diagnostic support was provided by 30.9% of clinics.
- Multidisciplinary reablement packages were provided by 9.1% (*n* = 5) of clinic respondents.
- Other areas (*n* = 55): 36.4% of clinics provide interventions for poor sleep-wake functioning. Furthermore, 36.4% of clinics offer psychological support and OT sessions, 30.9% provide speech and language assessments to improve and maintain communication abilities, and 27.3% provide psycho-education sessions.
- Referrals to services (*n* = 55): Most (92.7%) clinics refer clients for driving assessments, to Dementia Australia (89.1%), to speech pathologists (76.4%), and occupational therapists (70.9%). Less common are referrals to psychological support services and to local exercise groups or physiotherapists (58.5%), dieticians (54.5%), or legal services (45.5%).

Follow-up reviews (*n* = 55): Half of all clinics (51%) offer follow-up reviews to clients with an unclear diagnosis. Under half (47.3%) offer it to all clients or to those diagnosed with MCI (46.3%). Twenty clinics (36.4%) offer follow-ups to clients with an uncertain prognosis, while 18 (32.7%) in particular to those with complex identified issues that require multi-disciplinary expertise. Few clinics follow-up clients who were prescribed a cholinesterase inhibitor or memantine (23.6%) or those classified as having subjective memory complaints (16.4%).

No differences between metropolitan and regional or PUB-Cs and PRIV-Cs were observed in post-diagnostic practices, with the exception of sleep-wake interventions which were more commonly provided in metropolitan vs. regional clinics (45.0%, *n* = 18/40 vs. 13.3%, *n* = 2/15, respectively: χ^2^ = 4.7, *p* = .03). Also, compared to PUB-Cs, PRIV-Cs were more inclined to follow-up *all* clients (38.1%, *n* = 16/42 vs. 76.9%, *n* = 10/13, respectively:χ^2^ = 5.9, *p* = .01). Conversely, compared to PRIV-Cs, more PUB-Cs follow up clients with an unclear diagnosis (59.5%, *n* = 25/42 vs. 23.1%, *n* = 3/13, respectively:χ^2^ = 5.2, *p* = .02).

### Service improvements

- Out of 53 clinics, 96.2% expressed interest in utilising new blood-based biomarkers (e.g., pTau181) for accurate Alzheimer’s disease (AD) or preclinical AD detection, if rapid feedback were available. Conversely, when respondents were asked what percentage of their clients would agree to a lumbar puncture (where available, and costs minimal), of 49 respondents, 42.9% responded “none”, 46.9% responded “25 per cent”, 8.2% responded “50 per cent” and 2.0% responded “75 per cent”.
- In terms of ways to improve feedback, out of 19 respondents, who do not currently offer a separate feedback session, 73.7% indicated they would prefer to offer a separate feedback session if funding was available. The majority (87.4%) of valid respondents (*n* = 76/87) indicated they would like to provide an ‘automated’ dementia risk reduction report for people with subjective cognitive complaints or MCI. Of these 76 respondents, 94.7, 93.4 and 80.3% felt this should be provided to the general practitioner (GP), clients, and other referring medical practitioners, respectively, and 77.6% thought it should be provided to the family or carer. For provision of interventions, 95.9% (*n* = 93/97) felt that they would use a care plan resource if it could be auto-populated with local resources.
- To provide adequate post-diagnostic care, respondents (*n* = 86) felt that additional specialist clinicians or treatments would be required for the following areas: cognitive training (74.4%), exercise (62.8%), sleep (59.3%), diet (59.3%), counselling (57.0%), depression (43.0%), and other (10.5%, including speech and language therapy, addictions interventions, support groups, and outpatient integrated dementia-specific rehabilitation). All respondents (100%, *n* = 98) agreed that the client, as well as family or carer, should be involved in post-diagnostic care planning. The majority also agreed that: a) MCs must provide advice and referral to *other* services that provide post-diagnostic support (96.9%); b) all assessing clinicians should be actively involved in the client’s care planning (93.8%); and c) the client’s GP should be involved in the development and implementation of the care plan (88.8%).

## Discussion

This study describes Australian MC clinician and staff views on current practice. It highlights some key figures on wait-times for first appointments and differences in wait-times across public and private sectors, low rates of post-diagnostic support and some key differences across metropolitan and regional clinics.

Importantly, while MCs offer gold-standard multidisciplinary diagnostic services, only 23.8% of PUB-Cs can provide a non-urgent assessment within 2 months (40% for metropolitan clinics and 20% for regional clinics). This is consistent with our prior work, which showed the average wait-time is about 12 weeks in PUB-Cs [2]. Australian MCs’ wait-times are longer than those for MCs in Ireland (mean = 5.2 weeks, range 1-25 weeks, 19 clinics surveyed) [13], Canada (mean = 25 days) [14] and the United Kingdom (33 days in 2017-2018) [21].

We identified disparities between metropolitan and regional clinics. Metropolitan clinics were less likely to conduct an assessment of the client’s capacity to consent when compared to regional clinics. They also justified the need for neuropsychological assessments in a range of areas to a greater extent, which could reflect the greater availability of and funding for neuropsychologists in metropolitan areas. When compared to regional clinics, metropolitan clinics were five times more likely to discuss suitability for industry-sponsored clinical trials and to provide interventions for poor sleep-wake functioning. The disparities between metropolitan and regional areas are concerning, given that two of every five persons with dementia live in regional cities and towns or rural and remote communities of Australia [24]. Although this survey did not seek to understand the reasons behind the barriers contributing to the disparities in geographical service provision nor their solutions, further systems-level work is warranted to scope and address any service gaps.

While it is more common for disparities to exist between PUB-Cs and PRIV-Cs in developed countries such as Australia with a mixed-system of healthcare (i.e., Universal Medicare, plus optional private health insurance) [25], disparities between Australian PUB-Cs and PRIV-Cs are worth noting. Namely, PRIV-Cs were almost three times more likely than PUB-Cs to offer an initial appointment within one to 2 weeks for clients having an immediate assessment need, with a similar result observed for clients without an assessment need (within 2 months). It is noted however, that the assessment process itself may take longer in the private sector due to the need to refer for other multidisciplinary investigations, including neuropsychological services, medical tests or neuroimaging, which in turn, may be associated with significant wait-times. Future surveys may wish to capture overall time to diagnosis, which is an important priority area for clients. Indeed, prior data has reported that from the time of symptom onset to receiving a firm diagnosis of dementia was unacceptably high, around 3.1 years [17]. Hence, capturing delays into and between primary care and MCs are equally important as the time taken to access a MC diagnosis, once a referral is received.

There were no differences between PUB-Cs and PRIV-Cs regarding assessment and diagnosis and the provision of post-diagnostic care. Conversely, we observed differences for interdisciplinary case-conferences, neuropsychological assessment and interventions, and follow-up reviews. That is, PUB-Cs were more than twice as likely to discuss legal and financial capabilities compared to PRIV-Cs. Respondents from PRIV-Cs were three times more likely to agree that a neuropsychological assessment should be provided for every MC client, when compared to PUB-Cs. Lastly, PRIV-Cs were twice as likely to follow-up clients, compared to PUB-Cs. This again may reflect the need for PRIV-Cs to provide follow-up after other referrals and investigations have been completed, as opposed to PUB-Cs, which tend to provide assessments and referrals at one coordinated appointment.

The well documented specialist role of MCs has been further reinforced by developments in diagnostics and risk reduction strategies [26], as well as evidence-based models of post-diagnostic support and care [18, 27–31]. Both Australian (NHMRC Clinical Practice Guidelines) [32] and international dementia guidelines [33] emphasise the need for person-centred care, access to care coordination, specialised dementia care training for clinical staff, and community-based services to promote independence in activities of daily living. Most alarmingly however, we found that few Australian MCs are delivering evidence-based post-diagnostic care. While feedback and pharmacological support, carer and family support and management of depression is being provided by most MC care plans, other services such as advance care directives, telehealth, behavioural or cognitive interventions are inconsistently or rarely provided. While some of these services may be best delivered in the community or in primary care, there are some specialised interventions that MC clinicians are ideally positioned and qualified to deliver, if appropriately funded to do so.

For risk reduction, whilst 60% of respondents indicated they provide some degree of risk reduction education, most agreed that, they would utilise a standardised system for providing risk information to clients, family, and clinicians, if one were available. There are well-documented economic benefits of risk reduction [34], as well as knowledge increases [35] associated with the provision of risk reduction programs. Although preliminary work is emerging on communicating risk [36], effective methods to optimise delivery and behaviour change in relation to risk reduction strategies are not yet established [37].

For more targeted interventions that may improve cognition, there is now a Level I evidence base for the use of computerised cognitive training [38] and exercise training in MCI [39]. Moreover, a Level I evidence base has been established for strategy-based cognitive training for improving executive functions in older adults more generally [40]. However, the provision of these services in Australian MCs is very low. Less than 4% of MCs offered computerised cognitive training, and less than 10% of PUB-Cs offered memory strategy training. There are now at least two promising Australian models for such services [41, 42].

In terms of service improvements, 96.2% of clinicians expressed interest in utilising emerging blood-based biomarkers (e.g., pTau-181) to improve the accuracy of diagnosis for AD or preclinical AD. The majority of health professionals (87.4%) also felt that the communication of a patient’s dementia risk utilising an ‘automated’ tool, particularly for those living with MCI, could be helpful, if there were resources to support the efficient and effective use of such tools. Importantly, almost all health professionals (96.9%) agreed that MCs had a duty to appropriately refer clients to other post-diagnostic support services, and that all assessing MC clinicians should actively be involved in the client’s care planning (~ 94%).

Globally, the prevalence of undetected dementia is high, with estimated pooled rates being 61.7% [43]. Around 50% of dementia cases go undetected by GPs [17], with delays of more than 3 years between initial symptom presentation and formal diagnosis [17, 44]. Diagnoses in primary care are also unspecific, likely due to factors such as lack of sensitivity of tools, inadequate time [45], therapeutic nihilism, risk avoidance and concerns about competency and resources [46]. GPs need support from specialist MCs not only for diagnoses but also for management [47]. A variety of context specific solutions for addressing this need may be required including the use of electronic/telehealth/videoconferencing consultation (e.g., eConsult) [48], the use of GP ‘Champions’, team based care, the use of ‘care coordinators’ [49] and enablers for MC clinicians to provide ongoing training support as required.

Internationally, the number of MCs has been rising, which may in part reflect the rising cases of MCI and dementia, and increased recognition of the need for early diagnosis and care [50]. Notably, in 2002, the number of United Kingdom MCs tripled over a 10-year period [51]. In the Netherlands, MCs increased 8-fold from 1998 to 2016 with a concomitant 16-fold increase in new referrals [52], and a 10-fold increase in the number of patients diagnosed with dementia at a MC. In Australia, there has been a two-fold increase from the 23 clinics documented in 2009 [53], to an estimated 50 clinics in 2021 [54]. For a population of 17.3 M, there are 91 clinics in the Netherlands [52] (5.3 clinics per 1 M population). For Australia’s population of 25.8 M, the number of publicly funded MCs is currently estimated to be 50 [54] (1.9 clinics per 1 M population). Thus, overall, in comparison to international standards, Australia has not witnessed a similar increase in MC services.

International clinics have also witnessed a shift to diagnose earlier in the disease, and the proportion of patients with cognitive impairment without dementia has increased from 10 to 25% [52]. In Australia, we do not have an estimate of the annual number of MCI or dementia diagnoses made in MCs. A cross-sectional survey of an Aged Care Assessment Service in Western Australia suggested that 29% of incident dementia cases and 19% of prevalent cases are diagnosed in MCs [55], though notably these figures are influenced by the availability of other services such as private clinics. It is estimated that in 2025 there will be 318 new cases of dementia in Australia per day (*n* = 116,070 per year) [24]. Assuming the 50 PUB-Cs were to diagnose 29% of incident cases, each clinic would need to diagnose 673 cases per year. Our 2019 data showed that MCs provide services for an average of 3.3 new cases in 1 day [2]. Assuming each clinic was conducted 3 days per week for 48 weeks (a liberal estimate given that 10, 16 and 74% of clinics operate less than once per week, once per week, and more than once per week respectively) [2] - Australia would need to increase MC capacity by a minimum of 40% to meet the diagnostic demands alone by 2025 (i.e., not taking into account the need for such services to provide vital post-diagnostic support). Therefore, expansion of the MC network will be required and/or alternative solutions provided, such as Medicare or other infrastructure or funding support for private sector multidisciplinary services and increased expertise and support within primary care networks. Mapping of services in areas with large and diverse ageing and dementia populations will be particularly required, and in those regions with lower socioeconomic status.

### Limitations

There were several limitations to this study. Firstly, the current study was cross-sectional in nature, reporting the results from questions pertaining to current practice in 2020 and did not seek to examine changes in a health professional’s perceptions of services over time. It was also conducted during the SARS-CoV-2 (COVID-19) pandemic which may have influenced results. Second, while the number of clinicians working in MCs in Australia is currently unknown, the sampling method was not random and participants self-selected participation, thus it is unclear how representative the sample of respondents were in relation to all Australian MC clinicians. Indeed, the majority of MCs serviced ‘catchment areas’ of generally high socioeconomic status. It is unclear if this reflects greater provision of services in these regions or response bias from health professionals working in these areas. Third, the use of survey methods offers only approximate gauges of practice and does not produce data equivalent to electronic health records or national Medicare data extracts. With the release of the National Memory Clinic Service Guidelines [56], plans are underway to conduct an in-depth audit of clinics, and to align the Guidelines with the ADNeT Clinical Quality Registry [57]. Finally, it is important to note that this survey probed a number of topics that were considered important to develop National Memory Clinic Service Guidelines. Therefore, while is has breadth of coverage, future surveys may wish to focus on further in-depth analysis of particular areas.

## Conclusion

In conclusion, our data shows that in comparison to international standards, Australian MCs have not sufficiently increased to match the rise in dementia cases. Australian wait-times are excessively long, and post-diagnostic support or evidence-based strategies targeting cognition are rarely provided. In some aspects, there remains a disparity in services in regional versus metropolitan areas and publicly funded versus private clinics. ADNeT’s National MC Guidelines will provide service standards to address these issues. However, government investment will be required to implement best practice nationally.

## Supplementary Information

**Additional file 1.**

**Additional file 2: Supplementary Material 1**.

## Data Availability

A copy of the survey and the de-identified dataset analysed for the current study are available from the corresponding author on reasonable request.
