# Supplementary material for: Characterising Australian memory clinics: current practice and service needs informing national service guidelines
Source: BMC Geriatr. 2022 Jul 14;22:578. doi: 10.1186/s12877-022-03253-7 (PMC9281346; doi:10.1186/s12877-022-03253-7)
Supplement: Supplementary file 1 — Additional file 1. [file 12877_2022_3253_MOESM1_ESM.docx]

**Appendix A:**

| ADNeT Chief Investigators | |
| --- | --- |
| **ADNeT Chief Investigators** | **Principal Institution** |
| Christopher Rowe | The University of Melbourne |
| Perminder Sachdev | University of New South Wales |
| Sharon Naismith | University of Sydney |
| Michael Breakspear | The Council of the Queensland Institute of Medical Research |
| Henry Brodaty | University of New South Wales |
| Ralph Martins | Macquarie University |
| Stephanie Ward | University of New South Wales |
| James Vickers | University of Tasmania |
| Colin Masters | The University of Melbourne |
|  |  |
|  |  |
|  |  |
| ADNeT Associate Investigators | |
| **ADNeT Associate Investigators** | **Principal Institution** |
| Peter Schofield | Neuroscience Research Australia |
| Rob Grenfell | The Council of the Queensland Institute of Medical Research |
| Susan Kurrle | University of Sydney |
| Elizabeth Beattie | Queensland University of Technology |
| Ashley Bush | Florey Institute of Neuroscience and Mental Health |
| Maria Crotty | Flinders University |
| Annette Dobson | University of Queensland |
| Leon Flicker | University of Western Australia |
| Paul Maruff | The University of Melbourne |
| John McNeil | Monash University |
| Peter Nestor | University of Queensland |
| Olivier Salvado | University of Queensland |
| Susannah Ahern | Monash University |
| Nicole Kochan | University of New South Wales |
