# Supplementary material for: Characterising Australian memory clinics: current practice and service needs informing national service guidelines
Source: BMC Geriatr. 2022 Jul 14;22:578. doi: 10.1186/s12877-022-03253-7 (PMC9281346; doi:10.1186/s12877-022-03253-7)
Supplement: Supplementary file 2 — Additional file 2: Supplementary Material 1. [file 12877_2022_3253_MOESM2_ESM.docx]

**Supplementary Material 1:**

- Clinical assessments: The majority of clinics enquired about driving (98.3%, n = 58), family history (98.3%, n = 58), possible risk factors (96.6%, n = 57), presence of hallucinations/psychosis (96.6%, n = 56), social support (94.9%, n = 56), sleep (94.9%, n = 56), mobility and falls (94.9%, n = 56). Language abilities were assessed by 89.8% (n = 53) of clinics, while hearing and vision were assessed by 88.1% (n = 52) and 86.4% (n = 51) of clinics, respectively. There were no significant differences between the responses from the metropolitan/regional or the public/private clinics for topics covered >70% by clinicians.

The following topics were covered by <70% of clinics: nutrition (67.8%, n = 40), legal issues (66.1%, n = 39), client’s and family/carer’s expectations of assessment (64.4%, n = 38), client’s advanced care plans (59.3%, n = 35), exercise routine (55.9%, n = 33). Metropolitan and regional clinics differed on the topics of nutrition (60.5%, n = 26/43 *vs*. 87.5%, n = 14/16 respectively: χ^2^ = 3.8, *p* = .05). The coverage of legal issues differed for metropolitan (58.1%, n = 25/43) *vs.* regional (87.5%, n = 14/16)(χ^2^= 3.9, *p* = .049) and the public *vs.* private clinics (75.6%, n = 34/45 *vs.* 35.7%, n = 5/14, respectively: χ^2^ = 7.5, *p* = .01).

- Inter-disciplinary case conferences: 88.4% (n = 38/43) *of clinics* noted their use of a consensus diagnosis model. More than 75% of clinics reported discussing imaging results (86.0%, n = 37/43), driving capabilities (86.0%, n = 37/43), care strategies for the client (79.1%, n = 34/43), referral to allied health (external to the department) (76.7%, n = 33/43), and pharmacological intervention options (76.7%, n = 33/43). Significantly more PUB-Cs (94.3%, n = 33/35) used the consensus diagnosis model compared to PRIV-Cs (62.5%, n = 5/8) (χ^2^ = 6.3, *p* = .001), and were more likely to discuss legal and financial capabilities (74.3%, n = 26/35 *vs.* 25.0%, n = 2/8: χ^2^ = 6.8, *p* = .01). Compared to regional clinics (93.5%, n = 29/31), metropolitan clinics (66.7%, n = 8/12) were significantly more likely to discuss imaging results (χ^2^ = 5.0, *p* = .02) and pharmacological intervention options (87.1%, n = 27/31 *vs.* 50.0%, n = 6/12, respectively: χ^2^ = 6.5, *p* = .01). Less than 70% of total clinics reported discussing client’s strengths and resources (67.4%, n = 29/43), legal and financial capabilities (65.1%, n = 28/43), risk of abuse (65.1%, n = 28/43), medical prognosis (62.8%, n = 27/43), early support (62.8%, n = 27/43), risk factor modification (62.8%, n = 27/43), counselling (60.5%, 26/43) and educational needs (53.5%, n = 23/43), cognitive intervention options (46.5%, n = 20/43), suitability for investigator-led research studies (41.9%, n = 18/43) and industry-sponsored clinical trials (34.9%, n = 15/43). Compared to regional clinics (8.3% (n = 1/12), metropolitan clinics (45.2%, n = 14/31), were significantly more likely to discuss suitability for industry-sponsored clinical trials (χ^2^ = 5.1, *p* = .02).
